# A Spike Train Production Mechanism Based on Intermittency Dynamics

**DOI:** 10.3390/e27030267

**Published:** 2025-03-04

**Authors:** Stelios M. Potirakis, Fotios K. Diakonos, Yiannis F. Contoyiannis

**Affiliations:** 1 Department of Electrical and Electronics Engineering, University of West Attica, Ancient Olive Grove Campus, 12241 Egaleo, Greece; yiaconto@uniwa.gr; 2 Institute for Astronomy, Astrophysics, Space Applications and Remote Sensing, National Observatory of Athens, Metaxa and Vasileos Pavlou, Penteli, 15236 Athens, Greece; 3 Department of Physics, University of Athens, 15874 Athens, Greece; fdiakono@phys.uoa.gr

**Keywords:** spike train, artificial neural networks, biological neurons, intermittency, criticality, tricriticality, phase transitions

## Abstract

Spike structures appear in several phenomena, whereas spike trains (STs) are of particular importance, since they can carry temporal encoding of information. Regarding the STs of the biological neuron type, several models have already been proposed. While existing models effectively simulate spike generation, they fail to capture the dynamics of high-frequency spontaneous membrane potential fluctuations observed during relaxation intervals between consecutive spikes, dismissing them as random noise. This is eventually an important drawback because it has been shown that, in real data, these spontaneous fluctuations are not random noise. In this work, we suggest an ST production mechanism based on the appropriate coupling of two specific intermittent maps, which are nonlinear first-order difference equations. One of these maps presents small variation in low amplitude values and, at some point, bursts to high values, whereas the other presents the inverse behavior, i.e., from small variation in high values, bursts to low values. The suggested mechanism proves to be able to generate the above-mentioned spontaneous membrane fluctuations possessing the associated dynamical properties observed in real data. Moreover, it is shown to produce spikes that present spike threshold, sharp peak and the hyperpolarization phenomenon, which are key morphological characteristics of biological spikes. Furthermore, the inter-spike interval distribution is shown to be a power law, in agreement with published results for ST data produced by real biological neurons. The use of the suggested mechanism for the production of other types of STs, as well as possible applications, are discussed.

## 1. Introduction

**Figure 1:**
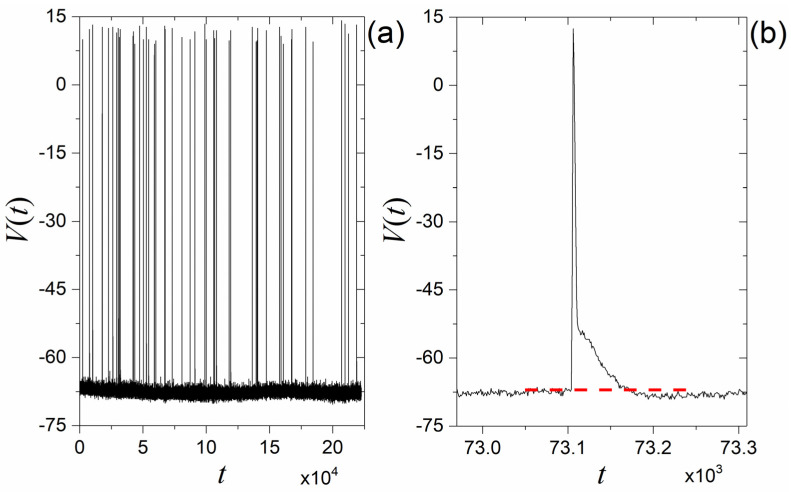
(**a**) Biological membrane potential $V\left(t\right)$ of neuron 089s1c1 from in vitro intracellular recordings of CA1 pyramidal neurons of Wistar male rats (adopted from [17]). (**b**) Zoom-in to a spike from Figure 1a, along with pre- and after-spike high-frequency fluctuations. The horizontal dashed red line, denoting the firing threshold, highlights the fact that the after-spike mean level is lower than the pre-spike one (hyperpolarization phenomenon).

Spike structures appear in the natural world in several phenomena, for example in astrophysical phenomena (diffraction spikes) [1,2,3], in laser devices [4,5,6], in biological nervous systems [7,8,9,10], and in biological structures, such as spike proteins and the coronavirus (e.g., COVID-19) [11,12,13,14,15]. Especially in the class of the dynamical-in-time spikes (spike train), spikes play a very important role because these structures carry information in time. The most typical example of a spike train (ST) is the action potential of biological neurons that carries the stimulus information in the biological neural network. The membrane potential of a biological neuron consists of two parts: (a) the action potential (voltage spikes), and (b) the relaxation time intervals (characterized by high-frequency voltage fluctuations). Figure 1a depicts a typical segment of a membrane potential time series, where the ST and the relaxation time intervals (high-frequency fluctuations) between the spikes are shown. Figure 1b presents a zoom-in to one of the spikes of Figure 1a, along with the pre- and after-spike high-frequency fluctuations. The experiment through which the specific membrane potential time series was acquired is described in detail in [16], whereas the time series was first presented and analyzed in [17]. Figure 1b is the same as Figure 3a of [17].

When the membrane potential reaches a threshold (denoted by the red line in Figure 1b), the biological neuron fires and generates a signal (spike). This is a temporal process. The individual spikes of Figure 1 present specific morphological characteristics. They present a sharp peak, a firing threshold and the biological phenomenon of the hyperpolarization; that is, the fall part of spike goes lower than the pre-spike relaxation values as in Figure 1b. Moreover, as shown in [16,17], the zone of high-frequency fluctuations between the spikes obeys dynamics that are met in the description of magnetization fluctuations near the critical point [16,18,19] (see also Section 2).

In the present work, we focus on STs of the biological neuron type, as in Figure 1. The main scope of this work is to investigate what mechanism could create such an ST. Several models have already been proposed that capture aspects of the biological neuron functioning or suggest simplified, computationally efficient alternatives, e.g., refs. [7,20,21,22,23]. However, these cannot reproduce the above-mentioned dynamics of the high-frequency fluctuations found in the relaxation intervals. This is important because it means that these cannot reproduce the spontaneous emergence of membrane fluctuations [16]. If there is really an ST production mechanism that could fill this gap, understanding it could help us design an artificial neural network (ANN) closer to the functioning of a biological neural network.

Although ANNs were inspired by the functioning of the nervous system (and brain) of living organisms, they have so far embodied a subset of biological neural systems’ features. Thus, despite their extended and particularly successful use in pattern recognition and machine learning in general (including the relatively recent example of deep learning), they need to be further developed to fully emulate biological neural systems functioning.

In this direction, in recent years, significant efforts have been made to enrich the ANNs with additional features of the biological neural systems, such as with the creation of STs. It is reminded that when the membrane potential reaches a threshold in biological neurons, the biological neuron fires and generates a (time-evolving) ST signal. Thus, the effort to introduce time into ANNs through the threshold mechanism has recently begun with the aim of creating spikes. An ANN which does not transmit information at each propagation circle but transmits information only when a certain threshold is crossed is called a spiking neural network (SNN) [20,24,25,26,27,28,29]. Until now, the most prominent SNN seems to be the leaky integrate-and-fire model [30]. After firing, the state variable is reset to a lower value (as in the biological neurons hyperpolarization phenomenon). Spike-based activation of SNNs is not differentiable, thus, making it difficult to develop gradient-descent-based training methods to perform error backpropagation [31,32]. One way to overcome this problem is to use the sigmoid function, which is differentiable, as an approximation of the Heaviside step function [17,33]. Various techniques have been developed in order to equip SNNs with improved learning capabilities, elevating computational efficiency [33,34,35,36,37]. However, learning mechanisms for SNNs, including SNNs that present inhibition, cannot yet be considered a solved issue [38].

Our approach to the topic of ANNs and the challenge of simulations as close as possible to biological reality is quite different from the predominant research directions in this field. First, in [16], we studied the biological neuron and showed that the high-frequency fluctuations which appear in the relaxation intervals follow the critical dynamics as expressed through the critical intermittency type I map (see Section 2). Then, we introduced the so-called Hybrid Model (HM) [17]. This model is based on key concepts of the stochastic Hopfield ANNs with an important difference: instead of using the classical Boltzmann probability to calculate the statistical weights that spread the information in the ANN from layer to layer, HM uses Fermi Statistics because this interprets the $\left\{+1,-1\right\}$ duality as fermions. The term “hybrid” highlights the mixing of ANN formalism and basic principles of Physics. Although HM succeeded in producing the fractal structure of inter-spike intervals and the dynamics of the high-frequency fluctuations found in the relaxation intervals, the morphology of individual spikes was not always close enough to that of real biological ones. HM spikes presented high fluctuations in spike heights and stepped morphology at the repolarization part (fall part), while some individual HM spikes presented flat tops.

Continuing in the above-presented direction, in the present work, we suggest a mechanism for the production of STs in the form of a rigorous formalism based on the phenomenon of intermittency. Thus, the aim of the work is to propose a mechanism that is capable of producing STs that resemble the biological ST shown in Figure 1, in terms of the key morphological characteristics of individual spikes, the fractal structure of inter-spike intervals, and the dynamics of the high-frequency fluctuations observed in the relaxation intervals.

The rest of the paper is organized as follows: Section 2 presents key information about intermittency, a critical intermittency type I map and a tricritical intermittency map, focusing on the fact that they behave as “complementary” repellors. Section 3 presents the proposed ST production mechanism based on the appropriate coupling of critical and tricritical intermittency. Its generalized form and the details on its operation using the abovementioned maps are presented in Section 3.1. Section 3.2 demonstrates its application through a numerical experiment that produces an ST whose individual spikes share key morphological characteristics with the real biological spikes of Figure 1. The dynamical and fractal characteristics of the produced ST are analyzed in Section 4 and proven similar to those of the real biological spikes of Figure 1. Section 4.1 focuses on the dynamics of the relaxation intervals’ high-frequency fluctuations, whereas Section 4.2 focuses on the fractal structure of inter-spike intervals. A discussion on the future further investigation of the proposed mechanism, its connection to biological neuron functions/processes and its possible applications is provided in Section 5. Finally, Section 6 summarizes the conclusions.

## 2. Intermittent Dynamics

Based on previous works [16,17], which demonstrate that the high-frequency amplitude fluctuations of the time intervals between spikes obey critical intermittency dynamics, we examine the idea that a mechanism for ST production can be proposed based on the dynamics of intermittency. The phenomenon of intermittency consists in the temporal alternation of regions of low amplitude fluctuations, called “laminar regions”, with regions, which demonstrate high amplitude fluctuations (bursts).

It has been demonstrated that the fluctuations of the order parameter in simulated thermal systems close to the critical point, which signals the transition from paramagnetic to ferromagnetic behavior in magnetic systems, are mathematically described by the critical intermittency type I map [18,19]:

(1) $$\phi_{n+1}=\phi_{n}+u_{1}\phi_{n}^{z_{1}}+\varepsilon_{n},$$

where $u_{1}>0$, $z_{1}>0$ and $\varepsilon_{n}$ is a uniform “noise”, $\varepsilon_{n}\in \left[-\varepsilon_{1},+\varepsilon_{1}\right]$. Note that in case that the exponent is not an integer, the nonlinear term should be used as $+u_{1}{\left|\phi_{n}\right|}^{z_{1}}$. The map of Equation (1) will hereafter be referred to as “map1”.

As shown in [18,19], if $\phi_{n}$ denotes the fluctuations of the order parameter, then the exponent $z_{1}$ is connected to the isothermal critical exponent δ, which characterizes the above-mentioned transition, with the relation:

(2) $$z_{1}=\delta +1.$$

**Figure 2:**
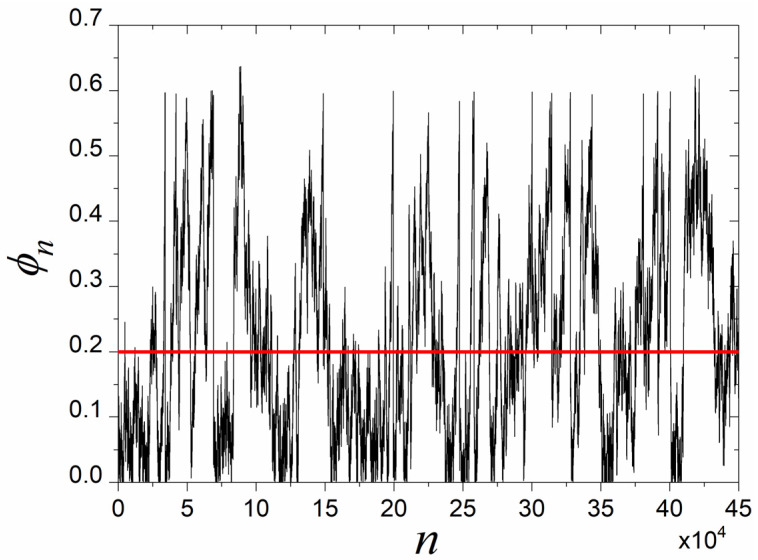
An intermittent time series in which it has been considered that the region from 0 up to 0.2 (bounded upwards by the red horizontal line) is the laminar region and all values above this zone correspond to bursts.

Figure 2 depicts a time series constructed using Equation (1) that presents the above-mentioned alternation between laminar regions and bursts. In Figure 2, the start of the laminar region is $\phi_{0}=0$, whereas the end of the laminar region has been considered to be $\phi_{L}=0.2$. This means that the time series remains within the laminar region as long as $\phi_{n}\in \left[\phi_{0},\phi_{L}\right]$; in this example it is $\phi_{n}\in \left[0,0.2\right]$. The way to construct a numerical simulation that produces an intermittent time series, as in Figure 2, is the following. One starts from a random value inside the laminar region. Each next value is calculated from the previous value using an appropriate recurrence relation, e.g., the intermittent map of Equation (1), until a burst ends. Then the trajectory randomly returns to the laminar region, and so on. A burst ends when the next value calculated by the recurrence relation exceeds a predefined high threshold, e.g., the value 0.65 for the time series of Figure 2. At this point, instead of using the calculated as the next value, a new value inside the laminar region, e.g., inside the interval $\left[0,0.2\right]$ for the time series of Figure 2, is used in its place. In this return mechanism, if the burst ends are considered as the input values, then the laminar region values are the output values. In this sense, the return from the burst region back to the laminar region is a kind of “feedback” mechanism.

In nature, many phenomena present intermittent dynamics [39], producing intermittent time series. In other words, intermittency presents universality. Could the universality of intermittency lead to a universal ST production mechanism? The waveform of a spike comprises a rise part and a fall part (see Figure 1b). The map of Equation (1) can produce the rise part, but what about the fall part?

It is known from the theory of critical phenomena [40] that there is a region where a second-order phase transition, characterized by critical behavior, meets a first-order one, characterized by an abrupt change. This is accomplished around the so-called Griffiths tricritical point. As has been shown in [41], the thermal fluctuations of the order parameter near this point are described by another intermittent map of the form:

(3) $$\phi_{n+1}=\phi_{n}-u_{2}\phi_{n}^{-z_{2}}+\varepsilon_{n},$$

where $u_{2}>0$, $z_{2}>0$ and $\varepsilon_{n}$ is a uniform “noise”, $\varepsilon_{n}\in \left[-\varepsilon_{2},+\varepsilon_{2}\right]$. Note that, as in Equation (1), in case the exponent is not an integer, the nonlinear term should be used as $-u_{2}{\left|\phi_{n}\right|}^{-z_{2}}$. The map of Equation (3) will hereafter be referred to as “map2”. The negative sign of the nonlinear term and the negative exponent ensure that the values fall. The reader should keep in mind that, according to the previous discussion, map1 can be called the critical intermittency type I map and map2 the tricritical intermittency map.

**Figure 3:**
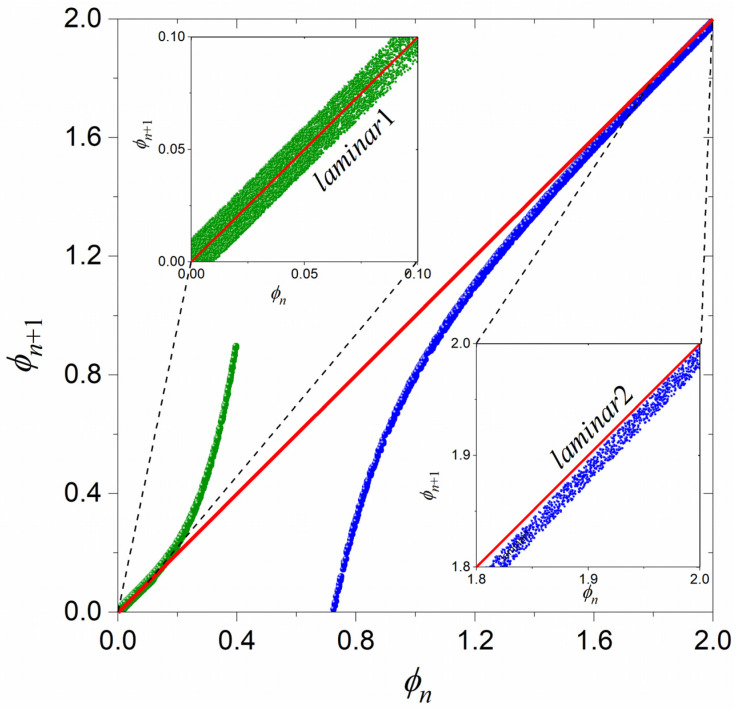
Typical examples of the return plots of the map1, given by Equation (1) (green), and the map2, given by Equation (3) (blue). The laminar region in both maps is the part of the trajectory that closely follows (is almost parallel to) the bisector (red line). A zoom-in to each laminar region, laminar1 and laminar2, is presented in the corresponding insets. As soon as the trajectory begins to move away from the bisector, the map has entered the bursts region. Such plots can be constructed for various combinations of the parameters’ values in Equations (1) and (3), moving the two laminar regions closer or further away from each other.

The two maps of Equations (1) and (3), i.e., map1 and map2, are repellors with respect to their fixed points. The fixed point of map1 is at zero (marginally unstable) and its nonlinear term leads the trajectory to higher values (rise), departing from its fixed point and allowing the trajectory to approach the fixed point of map2, which lies at a high value (theoretically infinity). Similarly, the fixed point of map2 is also marginally unstable and its nonlinear term leads the trajectory to lower values (fall), departing from its fixed point and allowing the trajectory to approach the fixed point of map1. Figure 3 presents an example of return plots of these two maps: on the left is the critical intermittency type I (map1) and on the right is the tricritical intermittency (map2). As can be seen from Figure 3, the nonlinear term ${u_{1}\phi }_{n}^{z_{1}}$ of map1 drives the trajectory away from the laminar region, marked as “laminar1” ($\phi_{n}\in \left[{\left(\phi_{0}\right)}_{1},{\left(\phi_{L}\right)}_{1}\right]$), by leading to higher values, moving it away from the bisector. This can be considered an “excitatory” process in biological neuron terms. On the contrary, the nonlinear term $-u_{2}\phi_{n}^{{-z}_{2}}$ of map2 drives the trajectory away from the laminar region, marked as “laminar2” ($\phi_{n}\in \left[{\left(\phi_{L}\right)}_{2},{\left(\phi_{0}\right)}_{2}\right]$), by leading to lower values, moving it away from the bisector. This can be considered an “inhibitory” process in biological neuron terms. In that sense, these two intermittent maps, map1 and map2, can be seen as “complementary” repellors.

## 3. Spike Train Production Mechanism Based on Intermittency Dynamics

### 3.1. Suggested Spike Train Production Mechanism

**Figure 4:**
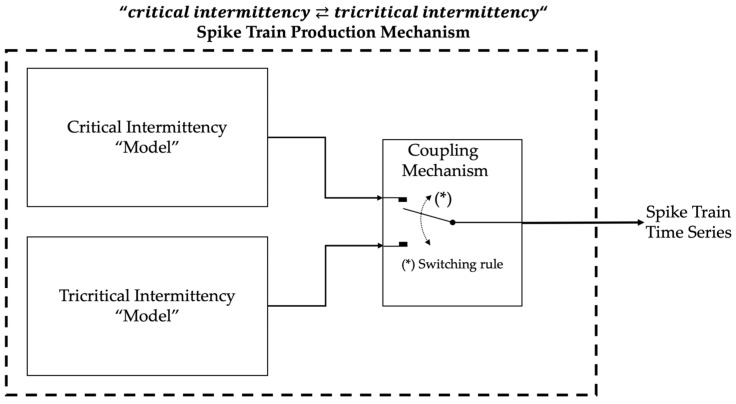
Suggested spike train production mechanism based on intermittency dynamics.

Based on the complementary repellor behavior of the critical and tricritical intermittent maps presented in Section 2, it is expected that an appropriate coupling of these two maps, which would allow their temporal alternation, “map1⇄map2”, each time one of the maps crosses a certain threshold value—$\phi_{Th1}$ (for map1) and $\phi_{Th2}$ (for map2)—at the end or outside the corresponding laminar region, could indeed lead to ST production. Generalizing this suggestion, an appropriate coupling of any critical intermittency “model” with any tricritical intermittency “model”, which would allow a corresponding “critical intermittency⇄tricritical intermittency” temporal alternation with the switching criterion being the crossing of the matching thresholds, could also lead to ST production (Figure 4). By the terms critical intermittency “model” and tricritical intermittency “model,” we mean any methods of generating time series that exhibit the corresponding dynamics, such as mathematical expressions, ANNs, or even appropriately using parts of real recorded time series.

Let us see how the suggested spike train production mechanism can be defined in detail, using map1 and map2 as critical intermittency and tricritical intermittency “models”, respectively.

As already mentioned, the “critical intermittency⇄tricritical intermittency” temporal alternation is performed with a switching criterion: the crossing of a threshold value at the end or outside the corresponding laminar region. For the “map1⇄map2” case, let us suppose that the procedure starts with map1. For map1, the initial conditions are a low value $\phi_{n=0}\in laminar1$ and $\varepsilon_{n=0}\in \left[-\varepsilon_{1},+\varepsilon_{1}\right]$. As long as map1 produces values $\phi_{n+1}\in laminar1$, the switch in Figure 4 remains at the upper position that connects map1 output to the system’s output. As soon as the trajectory crosses upwards the threshold $\phi_{Th1}$, i.e., as soon as a next value $\phi_{n+1=k}>\phi_{Th1}$ is calculated (and before this reaches the system’s output), this triggers the switch in Figure 4 to change to the lower position. At this position, the switch connects the system’s output to the map2 output, which starts at a high value $\phi_{k}\in laminar2$. As long as the next values produced by map2 remain within laminar2, i.e., as long as $\phi_{n+1\geq k}\in laminar2$ holds, the switch in Figure 4 remains at the lower position that connects the map2 output to the system’s output. As soon as the trajectory crosses downwards the threshold $\phi_{Th2}$, i.e., as soon as a next value $\phi_{n+1=m>k}<\phi_{Th2}$ is calculated (and before this reaches the system’s output), this triggers the switch in Figure 4 to change back to the upper position. At this position, the switch connects the map1 output to the system’s output, starting at a low value $\phi_{m}\in laminar1$ and so on.

The two thresholds, $\phi_{Th1}$ and $\phi_{Th2}$, controlling the switch operation and, consequently, the coupling mechanism, depend on the considered critical intermittency and tricritical intermittency “model” parameters as well as on the targeted ST. In the case of STs of the biological neuron type, $\phi_{Th1}={\left(\phi_{L}\right)}_{1}$, which is the end of the laminar region of map1, while $\phi_{Th2}={\left(\phi_{0}\right)}_{1}$, which is the fixed point of map1. It is noted that $\phi_{Th1}$ signifies the firing threshold and, therefore, the start of each spike. Setting $\phi_{Th2}={\left(\phi_{0}\right)}_{1}$ ensures that after each spike the trajectory returns to a value lower than the firing threshold, thus, reproducing the hyperpolarization phenomenon.

In the following, we will examine whether the suggested mechanism, using map1 and map2, can indeed produce an ST resembling a biological neuron ST, such as the one shown in Figure 1.

### 3.2. Spike Train Production Example

**Figure 5:**
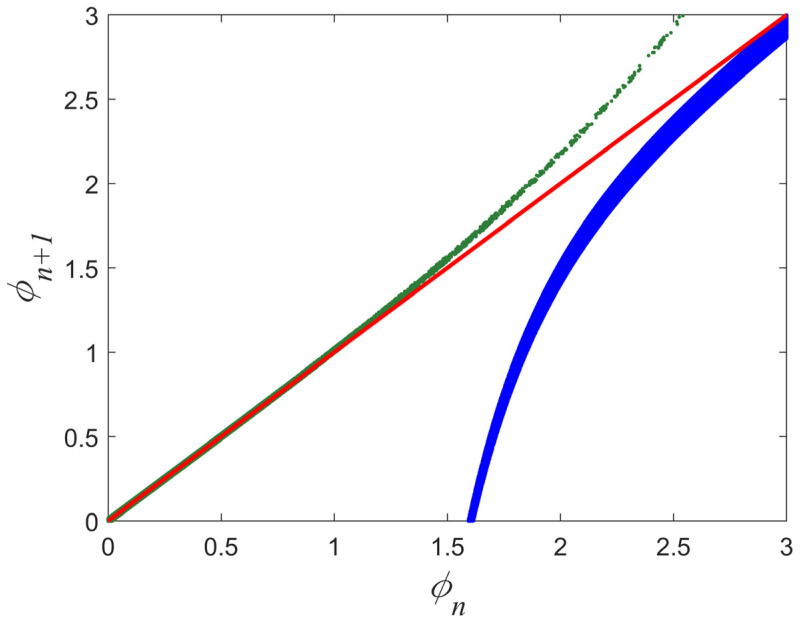
Return plots of map1 (green) and map2 (blue), uncoupled, for the maps parameters values used in our numerical experiment (see text). Red line denotes the bisector.

By setting specific values for all the parameters of map1 (Equation (1)) and map2 (Equation (3)), as well as for $\phi_{Th1}$ and $\phi_{Th2}$ (see Section 3.1), a numerical experiment can be performed that demonstrates the effectiveness of the coupling mechanism suggested in Section 3.1. A set of such values is (a) map1 {$z_{1}=4$, $u_{1}=0.011$, $\varepsilon_{1}=0.0175$}, (b) map2 {$z_{2}=5$, $u_{2}=17$, $\varepsilon_{2}=0.07$}, and (c) switching thresholds {$\phi_{Th1}=0.31$, $\phi_{Th2}=0$}. The specific $\phi_{Th1}$ and $\phi_{Th2}$ values are, respectively, the end of the laminar region and the fixed point of map1 for the above-mentioned map1 parameters, following the rule mentioned in Section 3.1 for the production of STs of the biological neuron type. The return plots of each map for the above-mentioned parameters, without any coupling between them, are shown in Figure 5.

In our example, the high value of the laminar region of map2 at which the trajectory starts upon switching to map2 has been set to be $\phi_{k}=3\in laminar2$. Thus, the switch from map1 to map2 means a jump to the value 3. Correspondingly, both the initial value and the low value upon switching to map1 have been set to be ${\;\phi }_{n=1}=\phi_{m}=0\in laminar1$, i.e., the fixed point of map1. Therefore, the switch from map2 to map1 means a jump to the value 0. Although in our experiment the values at which each map starts upon switching were fixed, the randomness in the coupling between the two maps is ensured by the random number generators, which are used to generate the “noise” terms $\varepsilon_{n}$ of map1 and map2 (see Equations (1) and (3)). Thus, each time the trajectory enters the laminar region of each map, a different path is followed due to the term $\varepsilon_{n}$ included in both Equations (1) and (3), and, consequently, different laminar lengths L, i.e., waiting times within the laminar region (see also Section 4.1), are produced. This randomness is enough and there is no need to introduce additional randomness at the entry points for each laminar trajectory. However, this certainly does not rule out sending the trajectory at a random point of the corresponding laminar region upon switching, which is not affecting the results.

**Figure 6:**
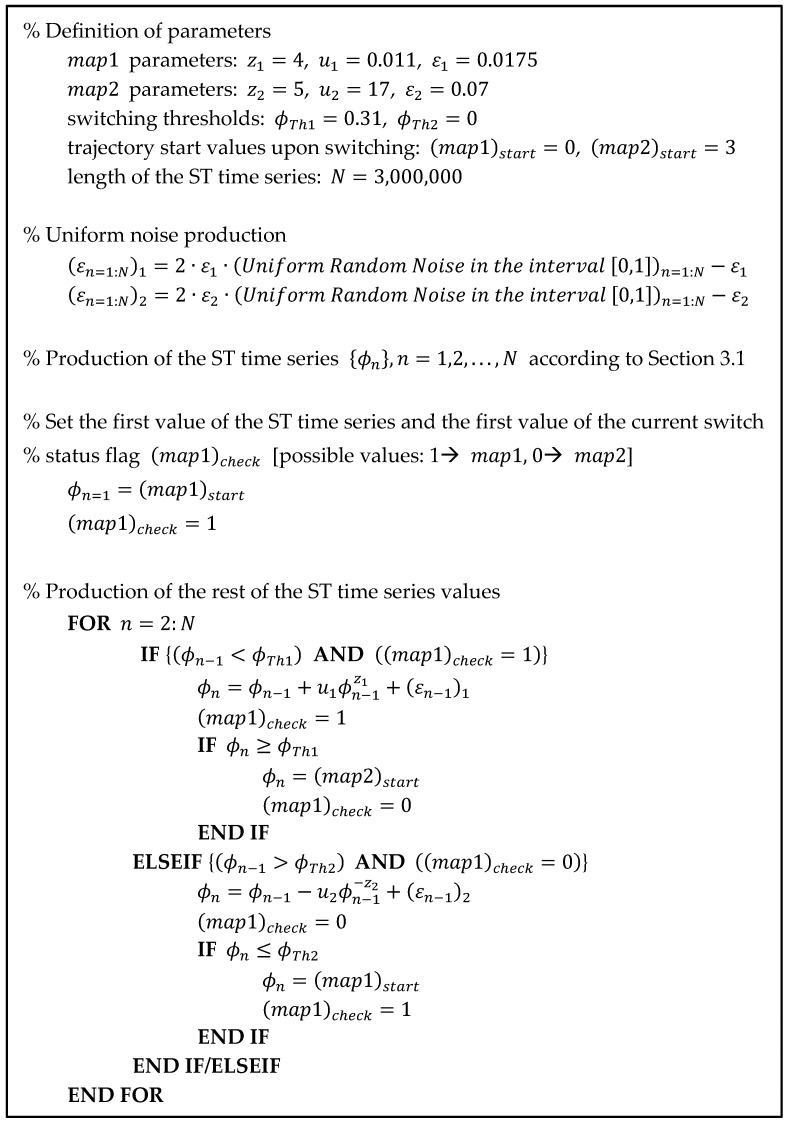
Pseudocode for the production of the ST time series presented in Figure 7.

**Figure 7:**
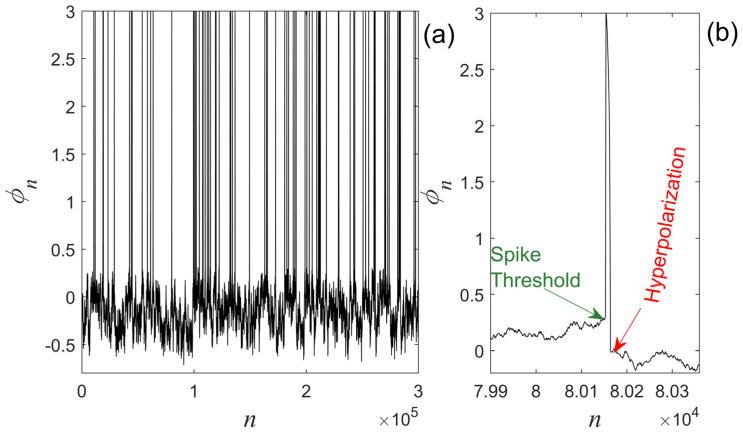
(**a**) The time series produced by the ST production mechanism suggested in Section 3.1, using the parameters: map1 {$z_{1}=4$, $u_{1}=0.011$, $\varepsilon_{1}=0.0175$}, map2 {$z_{2}=5$, $u_{2}=17$, $\varepsilon_{2}=0.07$}, switching thresholds {$\phi_{Th1}=0.31$, $\phi_{Th2}=0$}. Only the first 300,000 points of the produced 3,000,000-points-long time series are shown, so that the spike pattern is clearly visible. (**b**) A spike from the time series of Figure 7a where the spike threshold and the hyperpolarization phenomenon are marked.

The experiment was run until the algorithmic time n=N=3,000,000, i.e., until the length of the time series reaches a target value of N=3,000,000 points. The pseudocode that produces the ST time series is shown in Figure 6, whereas the Matlab v. 2024a source code for the numerical experiment is provided in the online available Supplementary Material of this article. The produced ST time series is shown in Figure 7a. It is noted that such a long length for the time series was selected for two reasons. Primarily, to achieve a population of spikes sufficient for inter-spike intervals statistical analysis (see Section 4.2). But also, to tighten the 95% confidence intervals (95%CI) of the estimated values during the analysis presented in Section 4.1 and Section 4.2.

As can be seen from Figure 7a, the time series produced by the mechanism suggested in Section 3.1 is an ST. From Figure 7b, it is clear that the generated spikes present sharp peaks, the spike threshold, and the phenomenon of hyperpolarization, which are signatures of biological neuron-type spikes (see Figure 1b). In our suggested ST production mechanism, the external excitation parameter (external field) is absent. The intermittency is an “internal” dynamic with feedback mechanisms between the region of smooth fluctuations (laminar region) and the region of strong fluctuations (bursts). Therefore, the imposition of external fields would destroy this internal self-organization.

The coupling of the two intermittent maps successfully generates STs of the biological neuron type, as they exhibit key characteristics of STs produced by biological neurons. Beyond the morphological resemblance of the individual spikes—sharp peaks, spike threshold, and hyperpolarization—, both the dynamics of the relaxation intervals fluctuations and the fractal nature of biological neuron STs are also reproduced by the suggested spike train production mechanism, as shown in Section 4.

## 4. Dynamical and Fractal Characteristics of the Produced Spike Train

### 4.1. On the Dynamics of the Relaxation Intervals’ High-Frequency Fluctuations

In this section, we study the dynamics of the relaxation intervals’ high-frequency fluctuations for the ST presented in Section 3.2, produced by the mechanism suggested in Section 3.1. In [16], it was found that the high-frequency fluctuations of Figure 1a for the biological neuron obey the critical dynamics as expressed through the intermittency map type I (map1, Equation (1)). The use of map1 in the suggested ST production mechanism serves the reproduction of this dynamics. This dynamics is determined from the distribution of the laminar lengths L (see below) of the intermittency. According to [39], for the map1, this distribution is a power law of the form:

(4) $$P(L)\sim L^{-p},$$

where p=z1z1−1. Due to Equation (2) we have that:

(5) $$p=1+\left(1/\delta \right).$$

Given the fact that 1<δ<∞, from Equation (5), it results that the critical intermittency exists for exponent values p∈[1,2). According to [41], the map2 laminar lengths’ distribution is also given by the Equation (4), where it is now:

(6) $$p=\frac{z_{2}}{z_{2}+1}=\frac{\delta +1}{\delta +2\;}.$$

Given the fact that 1<δ<∞, from Equation (6), it results that the tricritical intermittency exists for exponent values p∈[0.66,1).

The method of critical fluctuations (MCF) [18,19,42] is used for the estimation of the exponent p in a time series. According to MCF, it has been found [16] that the relaxation intervals’ high-frequency fluctuations of the biological neuron of Figure 1a obey critical intermittency with exponent p=1.37. This exponent is very close to the value 1.33, which results from Equation (5) for δ=3 and indicates the mean field theory (MFT) universality class of critical phenomena [40].

**Figure 8:**
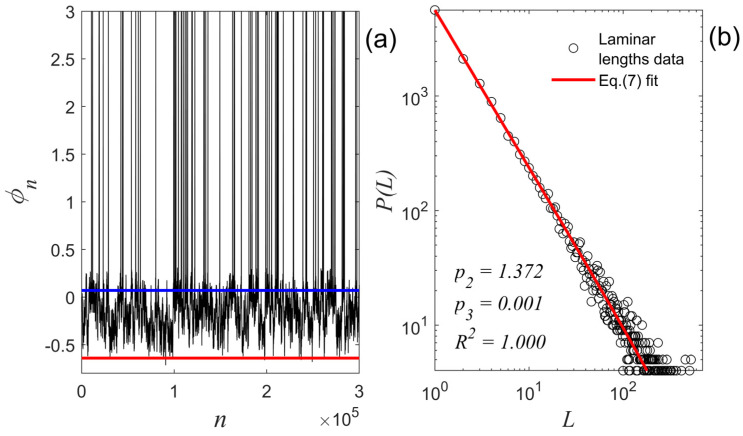
(**a**) The ST time series produced by the numerical experiment of Section 3.2 (also depicted in Figure 7a). The laminar region of the high-frequency fluctuations of the relaxation intervals was found to be bound between $\phi_{red}=-0.64$ (red horizontal line) and $\phi_{blue}=0.07$ (blue horizontal line). (**b**) The distribution of laminar lengths resulting from the laminar region marked in Figure 8a. The estimated exponent values by fitting Equation (7) are: $p_{2}=1.372$, $p_{3}=0.001$, with goodness of fit $R^{2}=1.000$. Although only the first 300,000 points of the analyzed time series are shown in Figure 8a, the distribution was calculated using the total length of the time series (3,000,000 points).

In the following, we apply the MCF to the relaxation intervals’ high-frequency fluctuations of the 3,000,000-points-long ST time series produced in the numerical experiment presented in Section 3.2. The application of MCF to an ST is demonstrated with the help of Figure 8. The red-colored level marked in Figure 8a denotes the lower value $\phi_{red}$ of the ST, whereas the blue-colored level denotes a higher value $\phi_{blue}:\;\phi_{red}<\phi_{blue}<\phi_{Th1}$ and is treated as a free parameter. The levels $\phi_{red}$ and $\phi_{blue}$ delimit a “laminar region” for the ST time series, which is actually a subset of laminar1 of map1. The laminar lengths L are the waiting times of the ST time series inside this zone. Thus, the laminar lengths are the number of successive ϕ-values obeying the condition $\phi_{red}<\phi <\phi_{blue}.$ As already mentioned, the $\phi_{blue}$ value is a free parameter. This means that all values $\phi_{red}<\phi <\phi_{Th1}$ are examined as possible $\phi_{blue}$ values, while the examination is performed exhaustively by progressively increasing the number of equally spaced values covering the whole amplitude range until the best power-law distribution of laminar lengths is found. As soon as the laminar lengths distribution is calculated, the following fitting function is used in order to estimate the power-law exponent:

(7) $$g\left(L\right)\sim L^{-p_{2}}e^{-p_{3}L}.$$

The specific fitting function is a truncated power-law function, where $p_{2}$ is the exponent of the power-law factor, and $p_{3}$ is the exponent of the exponential corrective term. If the estimated $p_{3}$ exponent is close to zero, the distribution tends to the scaling form of Equation (4); thus, $p_{2}$ is an estimate of the exponent p. As already mentioned, if p∈[1,2), then the time series has been produced by a system that is in its critical state. Therefore, the exponent $p_{3}$ is a measure of how close the system is to criticality if simultaneously $p_{2}\in [1,2)$.

We applied the fitting function of Equation (7) to the distribution of laminar lengths of Figure 8b, calculated for $\phi_{red}=-0.64$ and $\phi_{blue}=0.07$, and estimated the values of the exponents $p_{2}$ and $p_{3}$ by nonlinear least squares optimization using the trust-region-reflective algorithm [43]. We found $p_{3}=0.001$ with a $95\%\;CI:\left(-0.001,\;0.002\right)$ and $p_{2}=1.372$ with a $95\%\;CI:\left(1.364,\;1.381\right)$. Note that in this experiment, $\phi_{blue}=0.07$ is not the only $\phi_{blue}$ value leading to $p_{3}\approx 0$ (here considered as any $p_{3}\in \left[0,001\right]$). However, using any of these $\phi_{blue}$ values lead essentially to the same results. The obtained $p_{2}$ and $p_{3}$ exponents indicate the critical dynamics of the relaxation intervals’ high-frequency fluctuations of the ST produced by the mechanism suggested in Section 3.1. Moreover, the value of this exponent $p_{2},$ on the one hand, is very close to the theoretical one for the MFT (δ=3), which is p=1.33, and on the other hand, it is practically identical to the value of the exponent p=1.37 that has been determined in [17] for the membrane potential relaxation intervals’ high-frequency fluctuations of the biological neuron ST of Figure 1. Note that running the same numerical experiment again with different seeds for the pseudorandom number generators may lead to different $\phi_{blue}$ values that lead to $p_{3}\approx 0$. However, as soon as the $\phi_{blue}$ value leading to $p_{3}\approx 0$ is determined, the $p_{2}$ value, resulting from the application of the fitting function of Equation (7) to the distribution of laminar lengths, remains essentially the same.

### 4.2. On the Distribution of Inter-Spike Intervals

**Figure 9:**
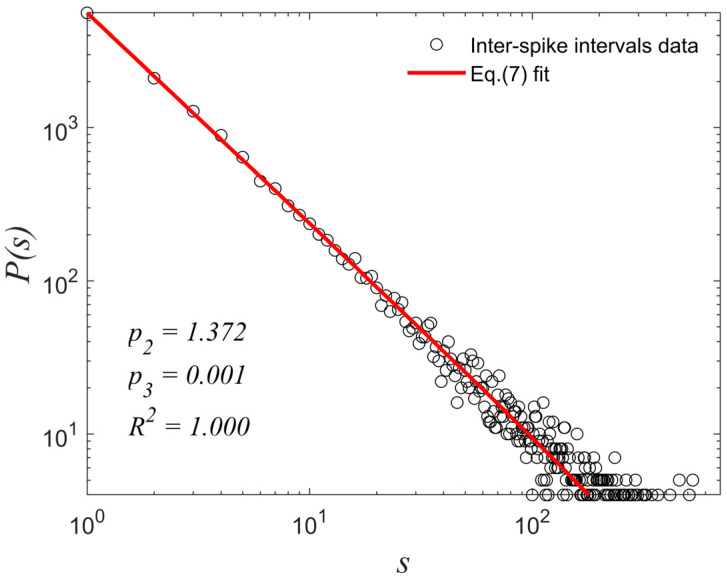
The distribution of the inter-spike intervals of the 3,000,000-points-long ST time series produced in the numerical experiment of Section 3.2 is a power law of the form $P(s)\sim s^{-1.372}$.

In this section, we calculated the distribution of the inter-spike intervals (distances between successive spikes) for the time series produced by the numerical experiment of Section 3.2. The distribution of inter-spike intervals for the 3,000,000-points-long ST time series is a power law of the form $P(s)\sim s^{-1.372}$ (Figure 9), proving the fractal structure of the portrait of the ST produced by the mechanism suggested in Section 3.1. This is yet another similarity to the STs produced by biological neurons, such as the one presented in Figure 1, which also exhibit a similar fractal structure [17]. It should be noted that the exponent of the inter-spike intervals power law is dependent on the selected parameters to conduct the numerical experiment. It is noted that the distribution was also fit to the truncated power-law function of Equation (7), also by nonlinear least squares optimization using the trust-region-reflective algorithm [43], and the result was identical to the fitting of the laminar lengths’ distribution of Figure 8b, i.e., $p_{3}=0.001$ with a $95\%\;CI:\left(-0.001,\;0.002\right)$ and $p_{2}=1.372$ with a $95\%\;CI:\left(1.364,\;1.381\right)$ with goodness of fit $R^{2}=1.000$.

## 5. Discussion

Criticality in nature is a universal phenomenon. Since critical dynamics are a key constituent element of the ST production mechanism suggested in Section 3.1, which is clearly reflected on the produced ST time series, it is expected that the suggested mechanism is universal. So, in future work, we intend to study additional critical and tricritical intermittency “models” (other than map1 and map2) within the framework of the ST production mechanism suggested in Section 3.1, both as alternatives to the production of STs of the biological neuron type and to investigate the production of other ST types found in different phenomena.

It has been shown in the pioneering work of Hodgkin et al. [7], that an ST, presenting the morphological characteristics of an ST produced by a biological neuron, can successfully be produced by the solution of the differential equations of an electrical circuit, known as the Hodgkin–Huxley model. As already mentioned in the Introduction Section, several other models have been proposed since its introduction, trying either to capture aspects of the biological neuron functioning or to suggest simplified, computationally efficient alternatives [20,21,22,23]. However, these cannot reproduce the dynamics of the high-frequency fluctuations found in the relaxation intervals [16,17]. But why does the coupling of the two types of dynamic intermittency succeed in producing STs that exhibit similarity to biological neurons in terms of the morphology of individual spikes, the power-law distribution of the inter-spike intervals and the dynamics of the relaxation intervals’ high-frequency fluctuations? Can this coupling of critical and tricritical intermittency be attributed to biological neuron functions/processes and under which conditions? The answer to these questions is more of a neuroscience problem and, thus, beyond the scope of the present work. However, we would like to propose, as a working hypothesis, that the two types of dynamic intermittency may be “hidden” in the sodium–potassium pump mechanism and possible feedback mechanisms between ionic transportation through the membrane and the membrane potential under specific conditions [17].

It has been suggested that biological neuron ST patterns serve as a kind of time encoding to pass messages across the nervous system and brain [44], while several efforts have been made to mimic this encoding with artificial systems, e.g., Refs. [44,45,46], as well as to achieve brain–machine interface [47] and to exploit it for cryptography applications, e.g., Refs. [48,49,50]. The STs produced by the mechanism proposed in Section 3.1 can produce STs with a variety of ST patterns, depending on the intermittency “models” parameters (e.g., map1 and map2 parameters), switching rules and pseudorandom number generator parameters. Thus, the proposed mechanism may be useful as an alternative for encryption and cryptography applications.

## 6. Conclusions

The findings of the present work prove that a coupling between the critical intermittency and the tricritical intermittency can lead to the production of STs. The individual spikes of the produced STs present the key morphological characteristics of biological spikes (sharp peak, spike threshold and hyperpolarization phenomenon). Moreover, the inter-spike interval distribution is a power law, whereas the high-frequency fluctuations found in the relaxation intervals present critical dynamics that belong to the Mean Field Theory universality class. The universal character of the phenomenon of intermittency implies that the suggested ST production mechanism may be able to produce STs that appear in different phenomena, as well as that different critical and tricritical intermittency “models” (other than map1 and map2) may be able to produce similar STs within the framework of the suggested mechanism. In future works, we intend to study various critical and tricritical time series generators, or real-world time series, in place of the critical and tricritical “model” blocks of the suggested ST production mechanism, with the aim of producing both the biological type and other types of STs to test mechanism’s universality.

## Data Availability

The original contributions presented in this study are included in the article. Further inquiries can be directed to the authors.

## Supplementary Materials

The following supporting information can be downloaded at: https://www.mdpi.com/article/10.3390/e27030267/s1, Matlab source code for the production of the ST time series.
