# Supplementary material for: A Spike Train Production Mechanism Based on Intermittency Dynamics
Source: Entropy (Basel). 2025 Mar 4;27(3):267. doi: 10.3390/e27030267 (PMC11941400; doi:10.3390/e27030267)
Supplement: Supplementary file 1 [file entropy-27-00267-s001.zip › entropy-3447723-supplementary.pdf]

entropy

Supplementary Material of the article

A Spike Train Production Mechanism Based on Intermittency Dynamics

Stelios M. Potirakis, Fotios K. Diakonos and Yiannis F. Contoyiannis

In the following, a Matlab source code for the production of the ST time series, as well as for the calculation of the laminar lengths and the inter-spike intervals is provided:

```
%% Matlab code start

close all; clear all; clc

%% Define parameters' values
% map1 parameters
z1 = 4;
u1 = 0.011;
e1 = 0.0175;

% map2 parameters
z2 = 5;
u2 = 17;
e2 = 0.07;

% Switching thresholds
phi_th1 = 0.31;
phi_th2 = 0;

% Trajectory start values upon switching
map1_start = 0;
map2_start = 3;

% Other constants definition / Initializations
nit = 3000000;              % Time series length
l = 0;                      % Counter for laminar length region
map1check = 1;              % Flag showing which map is currently selected
phi(1) = map1_start;        % First time series value

% "Laminar lengths" of the relaxation intervals’
% high-frequency fluctuations of the ST time series
% parameters / variable initializations
l = 0;                  % Laminar length counter
L = [];                 % Laminar lengths vector
phi_red = -0.64;        % Start of laminar lengths
phi_blue = 0.07;        % End of laminar lengths
```

```
%% Generation of pseudorandom numbers
rng(284,"twister")
en1 = 2* e1 * rand(1,nit) - e1; % Random perturbation for phi(i) of map1
rng(0,"twister")
en2 = 2* e2 * rand(1,nit) - e2; % Random perturbation for phi(i) of map2


%% Function map1

function x1ip1 = map1(x1i,u1,z1,en1)

% calculate next value based on Eq.(1)
x1ip1 = x1i + u1 * x1i^z1 + en1;

end


%% Function map2

function x2ip1 = map2(x2i,u2,z2,en2)

% calculate next value based on Eq.(3)
x2ip1 = x2i - u2 * x2i^(-z2) + en2;

end


%% Calculation of Spike Train time series

for j = 2:nit
    if phi(j-1) < phi_th1 && map1check == 1
        phi(j) = map1(phi(j-1),u1,z1,en1(j));
        map1check = 1;
        if phi(j) >= phi_th1
            phi(j) = map2_start;
            map1check = 0;
        end
    elseif phi(j-1) > phi_th2 && map1check == 0
        phi(j) = map2(phi(j-1),u2,z2,en2(j));
        map1check = 0;
        if phi(j) <= phi_th2
            phi(j) = map1_start;
            map1check = 1;
        end
    end
end


%% Count "Laminar lengths" of the relaxation intervals'
% high-frequency fluctuations of the ST time series
```

```
for i = 1:nit
    if phi(i) <= phi_blue && phi(i) >= phi_red
        l = l + 1;
    elseif l>0
        % Store the count of laminar lengths to a vector
        L = [L,l];
        % Reset laminar length counter
        l = 0;
    else
        l = 0;

    end
end


%% Count inter-spike intervals

stpks = find(phi==map2_start);
inter_spike_intrvl = diff(stpks);


%% Laminar lengths distribution calculation

edges = 0.5:1:max(L)+0.5;
bin_centers = (edges(1:end-1)+edges(2:end))/2;
[N,edges] = histcounts(L,edges);


%% Inter-spike intervals distribution calculation

edges_ispk_intrvl = 0.5:1:max(inter_spike_intrvl)+0.5;
bin_centers_ispk_intrvl = (edges_ispk_intrvl(1:end-1)+edges_ispk_intrvl(2:end))/2;
[N_ispk_intrvl,edges_ispk_intrvl] = histcounts(L,edges_ispk_intrvl);

%% Matlab code end
```
